# The effect of nine-year nitrogen addition on foliar fungal diseases of plant groups in an alpine meadow

**DOI:** 10.3389/fpls.2026.1899151

**Published:** 2026-09-18

**Authors:** Lingling Xu, Peng He, Longhai Xue, Chunjie Li

**Affiliations:** 1 State Key Laboratory of Herbage Improvement and Grassland Agro-ecosystems, Center for Grassland Microbiome; Engineering Research Center of Grassland Industry, Ministry of Education, Gansu Tech Innovation Center of Western China Grassland Industry, College of Pastoral Agriculture Science and Technology, Lanzhou University, Lanzhou, China; 2 Grassland Research Center of National Forestry and Grassland Administration, Institute of Ecological Protection and Restoration, Chinese Academy of Forestry, Beijing, China; 3 Institute of Agricultural Resources and Environment, Sichuan Academy of Agricultural Sciences, Chengdu, China

**Keywords:** alpine meadow, community structure, foliar fungal disease, nitrogen deposition, plant groups, plant-pathogen interaction, Qinghai-Tibet Plateau

## Abstract

**Introduction:**

Foliar fungal diseases have become one of the non-negligible drivers of accelerated grassland degradation, and global change processes such as increased nitrogen (N) deposition can significantly regulate the occurrence and development of foliar fungal diseases in meadows. However, little is known about how increased nitrogen deposition affects foliar fungal diseases in alpine meadows, and there is limited understanding of the relative importance of different plant groups in the process of N enrichment affecting plant diseases.

**Methods:**

Here, based on a 9-year continuous nitrogen addition experiment (2014-2022, gradients: 0, 2, 4, 8, 16, 32 g N m^-2^ year^-1^) in an alpine meadow on the Qinghai-Tibet Plateau, we investigated the effects of long-term N enrichment on plant group attributes and foliar fungal diseases through a one-time field survey conducted in August 2022. We employed linear mixed-effects models and structural equation modeling to explore the relationships between foliar fungal diseases and plant community variables under N enrichment.

**Results:**

We found that plant groups identity was the dominant factor influencing foliar fungal diseases, with Gramineae consistently exhibiting the highest disease incidence and disease index across all N addition levels. The SEM revealed that N addition did not exert a significant direct effect on disease index; rather, its effect was fully mediated by disease incidence-N addition significantly increased incidence rate, which in turn drove disease index. In addition, foliar fungal diseases tended to infect plants with greater relative coverage, especially Gramineae and Forbs, potentially because denser canopies promote pathogen spread and infection.

**Discussion:**

Overall, our results suggest that plant group composition plays a dominant role in determining foliar fungal disease patterns, with Gramineae being the most affected group, and that N addition influences disease development primarily by increasing disease incidence rather than by directly exacerbating lesion severity. These findings highlight the importance of considering foliar fungal disease occurrence in response to global change and the utility of plant groups as predictors of disease risk under N enrichment.

## 1 Introduction

The Qinghai-Tibet Plateau is the highest and widest alpine region on the earth, with a unique plant growth environment that makes ecosystem functions very sensitive (Jing et al., 2015). It functions as a regulator and indicator of environmental and climate changes on a global scale (Sun et al., 2018). Global climate change, irrational land use, and other factors have disrupted the equilibrium mechanisms of grassland ecosystems. This has resulted in a steady decline in grassland ecological balance and a persistent occurrence of diseases, which have a detrimental effect on the abundance and vigor of grassland plants and endanger the stability and functionality of the ecosystem as a whole (Fisher et al., 2012; Parker et al., 2015; Zhang et al., 2025). Of these, increased N deposition as a global change factor that is receiving a lot of attention can significantly impact plant diversity and foliar fungal disease in N-limited grasslands (Stevens et al., 2004; Ke et al., 2023; Zhang and Wang, 2023). Foliar fungal disease incidence in grasslands is the most prevalent and has emerged as a non-negligible factor in hastening grassland degradation (Allan et al., 2010; Liu et al., 2022).

In recent years, plant-microbe interactions have attracted significant interest from the scientific community (Kavamura et al., 2020; Latz et al., 2021; Pérez-Jaramillo et al., 2018; Xiao et al., 2024), and more than 19,000 species of fungi have been identified as causal agents of plant diseases worldwide. Foliar fungal diseases significantly influence the structure of plant communities by affecting the density, distribution, and genetic diversity of host plant populations, causing population declines and extinctions, thus disrupting the structural stability of plant communities and ecosystem functions (Fernández-González et al., 2020; Hantsch et al., 2013; Voriskova and Baldrian, 2013). The susceptibility of a plant to pathogen infection is influenced not only by its resistance to pathogen invasion and tolerance to infection but also by other factors, including the characteristics of the host plant (Liu et al., 2019; Spagnoletti et al., 2018). At present, many studies have been conducted on how foliar fungal diseases affect grassland ecosystems (Allan et al., 2010; Grunberg et al., 2023; Liu et al., 2022; Parker and Gilbert, 2018). In particular, decreased species richness and abundance in grasslands have been shown to be associated with increased disease susceptibility and severity (Mitchell et al., 2002). Potentially, reduction in plant species richness leads to reduced competition between species, which promotes the spread of diseases specific to that species within the community (Ampt et al., 2018; Karunarathna et al., 2021). Research on foliar fungal disease has focused predominantly on crops, such as corn and wheat (Chen et al., 2023; Latz et al., 2021; Lin et al., 2022), while studies on grassland foliar fungal disease are relatively scarce and limited. These studies focus still on artificial and semi-natural grassland systems, which are characterized by limited host species diversity, intensified management practices, and a relatively homogeneous ecological composition (Xue et al., 2020; Xu et al., 2024). The evaluation of foliar fungal diseases in natural grasslands faces challenges due to intricate plant-pathogen interactions and the competition and a wide range of traits among different hosts. However, research on foliar fungal diseases within the highly diverse Tibetan Plateau region is currently limited by geographical differences, climate, and grassland types (Grunberg et al., 2023; Mitchell et al., 2003). More research in this field is required to gain a more comprehensive understanding of the impacts of disease on natural grassland ecosystems.

Nitrogen (N) is an essential nutrient for plant growth and plays an important role in plant development and morphogenesis. It is also a key factor in limiting plant growth (Craine et al., 2003) and regulating community structure and function in terrestrial ecosystems (Mueller et al., 2015). N promotes the accumulation and growth rate of plant biomass while decreasing disease resistance. Furthermore, plants growing in nitrogen-rich conditions can exhibit higher foliar N concentrations, which potentially exacerbates their susceptibility to infections (nitrogen disease theory), leading to an increased incidence of fungal infections in plant foliage (Liu et al., 2019). Furthermore, the diversity of the plant community plays an important role in modulating the interaction between N addition and the incidence of foliar fungal diseases. N addition can directly or indirectly affect disease incidence (Veresoglou et al., 2013), transmission, and epidemiology (Clark and Tilman, 2008; Dawson et al., 2012) through its impact on plant community structure. The appearance of foliar fungal diseases can be effectively mitigated in highly diverse ecosystems through the “dilution effect.” However, increased N availability and exogenous addition have led to reductions in biodiversity (Han et al., 2020; Hunt et al., 2009; Midolo et al., 2019), weakening the dilution effect of host species richness on disease severity.

Despite extensive research on the impacts of N addition on ecosystems, substantial knowledge gaps persist concerning shifts in plant communities and disease susceptibility across different plant groups under N enrichment. Furthermore, there is still a lack of field evidence for differences in responses to fungal diseases of different plant groups caused by N addition. Here, we investigate the incidence and severity of foliar fungal diseases and the composition of natural grassland plant communities in an alpine meadow region under six gradients of N addition to address the following hypotheses: 1) Do different functional plant groups exhibit unique sensitivity and response patterns to fungal diseases induced by N supplementation; 2) Can the community structural characteristics of natural grassland plant communities serve as potential indicators for predicting the occurrence of foliar fungal diseases?

## 2 Materials and methods

### 2.1 Site and experimental description

The study was conducted in an experimental site managed over a nine-year period, which is part of the global change control experimental platform at the Sichuan Zoige Alpine Wetland Ecosystem National Observation and Research Station (https://regmarsh.Swun.edu.cn), located on the southeastern edge of the Qinghai-Tibetan Plateau, in Hongyuan County, Sichuan Province, China (32°48′ N, 102° 33′ E). Our work utilized the long-term nitrogen addition experiment led by researcher Niu Shuli, it is located at an altitude of approximately 3,500 meters (m), and it belongs to the continental plateau cold temperate semi-humid monsoon climate zone. The mean annual temperature is 1.5 °C, and the annual precipitation is 753 mm. The precipitation distribution is uneven, predominantly occurring from May to September, accounting for 80% of the total annual precipitation. The dominant species in this alpine meadow grasslands are *Deschampsia caespitosa*, *Carex schneideri*, *Kobresia setchwanensis*, and *Anemone rivularis*, collectively representing more than 90% of vegetation cover. The type of soil is alpine meadow soil, with a surface soil bulk density of 0.89 g cm^-3^ (Ma et al., 2018; Quan et al., 2024).

The detailed experimental design is described in Song et al. (2017) and Ma et al. (2018) as follows: the experiment employed a randomized complete block design that evaluated six addition rates of N (0, 2, 4, 8, 16, and 32 g N m^-2^ year^-1^), denoted as N0 (control), N2, N4, N8, N16 and N32, respectively. Each treatment was carried out in 5 replicates, resulting in a total of 30 regular 8×8 m plots, with a spacing of 3 m between the adjacent edges. During the 2014–2022 growing seasons (May-September), N was applied as NH_4_NO_3_ (> 99%) by hand every month before rainfall (Ma et al., 2020). To achieve an even distribution of NH_4_NO_3_ in the plots, dry NH_4_NO_3_ was thoroughly mixed with a small amount of local soil as a carrier and then surface-broadcast evenly. Finally, the experimental site was enclosed by guardrails to ensure accurate management practices.

### 2.2 Investigation of communities

This study was carried out on a long-term nitrogen addition experimental platform established in 2014, with nine years of nitrogen treatments completed by 2022. We conducted foliar disease surveys alongside overall vegetation community investigations in August 2022, during late August, one 1 × 1 m plot was established in each 8 × 8 m plot to investigate the plant communities. To mitigate edge influence, each plot was placed at least 1 m away from the edge as far as possible. All measurements were carried out using a quadrilateral frame (10×10 cm) randomly placed on each plot. The coverage of each plant was assessed using the acupuncture method and the number of plant species (plant species richness) within the plot was documented. For each plant species in the frame, 10 individual plants were randomly selected to measure their natural growth. Following the widely adopted classification framework in grassland ecology, plant species are commonly divided into four plant groups: Gramineae, Cyperaceae, Leguminosae, and Forbs (Wu et al., 2024; Zhang et al., 2021b). This framework, though based primarily on taxonomic affinity and growth form rather than on measured functional traits, is widely adopted for community-level analyses in alpine meadow ecosystems. Since no leguminous species were recorded in any of our 30 sampling plots, we adapted this framework to our field conditions and classified all plants into three groups: Gramineae, Cyperaceae, and Forbs.

### 2.3 Investigation of foliar fungal diseases

The incidence rate of the disease and the disease index have been widely recognized and applied in numerous disease investigations (Qiao et al., 2024; Zhang et al., 2019). Specifically, in this study, the incidence rate was defined as the percentage of plants or organs affected by the foliar fungal pathogen in each sample plot to the total number of plants examined. The disease index is a comprehensive index that considers the incidence rate and the severity of the disease and can be expressed as a representative graded value, the severity of fungal disease was quantified by calculating the proportion of the area of diseased plant organs (such as stems, leaves, flowers, fruits, etc.) to the total area of the corresponding organs of their host plants, the calculation takes into account the number of affected leaves at varying severity levels. The calculation formula is the following. Disease index = 100 × ∑(number of diseased leaves at each level × representative value at each level)/(total number of surveyed leaves × highest representative value). The grading of foliar disease severity is mainly based on the ratio of the area of the disease lesion to the leaf area: grade 0, without symptoms; grade 1, lesion area <5%; grade 2, lesion area 6% to 20%; grade 3, lesion area 21% to 40%; grade 4, lesion area 41% to 70%; and grade 5, lesion area >71%.

For each species within each plot, 50 leaves were randomly selected to assess incidence rate and disease index, if a species had fewer than 50 leaves, all available leaves were examined (Liu et al., 2019). Foliar disease evaluations were conducted on the 25 plant species recorded in this study. In addition, all symptomatic plants exhibiting leaf spot symptoms were collected and returned to the laboratory for isolation and identification of the associated fungal pathogens causing foliar disease symptoms (**Supplementary Tables S1**, **S2**).

### 2.4 Pathogen isolation and identification

Tissue blocks (1×0.5 cm) were excised from the junction between diseased and healthy tissue, then surface sterilized in 75% ethanol for 30 s, rinsed in sterile distilled water three times, and the sterilized tissue pieces were transferred to potato dextrose agar (PDA) medium. Plates were incubated at 25 °C in the dark for 3–7 days, with daily observation for fungal colony emergence. Emerging fungal hyphae were transferred to fresh PDA plates and repeatedly subcultured until pure cultures were obtained. Fungal identification was performed using a combination of morphological and molecular approaches. Pure cultures were examined under a compound light microscope (Olympus BX63, Tokyo, Japan), identification was based on colony characteristics (color, texture, growth rate), mycelial morphology, and reproductive structures (appressoria, seta, conidia, or ascospores), morphological features were compared with standard taxonomic references and fungal identification keys. Genomic DNA was extracted using an Omega Fungal DNA Extraction Kit (CA). The internal transcribed spacer (ITS) region of the ribosomal DNA was amplified using the universal primers ITS1 (5’-TCCGTAGGTGAACCTGCGG-3’) and ITS4 (5’-TCCTCCGCTTATTGATATGC-3’), and sequencing was performed by Tsingke Biological Technology (Xi’an, China). The resulting sequences were compared against the NCBI GenBank database using BLAST searches to determine the closest matched taxa. Species identification was accepted when sequences showed ≥ 99% similarity with reference sequences of known fungi, the identification of each isolated strain was detailed in **Supplementary Table 1**.

### 2.5 Statistical analysis

Community-level disease incidence and disease index were calculated as the arithmetic mean of species-level values per plot. To examine the relationship between N addition rate and disease severity, we fitted a linear model. To test for non-linearity, N addition levels were treated as a categorical factor; the non-significant deviation from linearity justified the use of a linear function. SPSS 20.0 was used to perform a one-way analysis of variance (ANOVA) to assess the significance of differences in the disease index and the incidence rate of the plant community under different rates of N addition. Tukey’s HSD test was used to compare the effects of different rates of N addition on the disease incidence rate and disease index in all communities and between the three plant groups. The same procedure was applied to compare disease metrics among the three plant groups. Linear regression analyses were conducted in Origin 2021 to examine the relationships between foliar disease metrics and community factors (relative coverage, species richness, and average height). General linear mixed-effects models (GLMMs) were used to evaluate the addition of N, plant groups, and their interactions on the severity of foliar fungal disease and community factors, with ‘block’ as a random effect. These general linear mixed-effects models were built using the lmer function in the lme4 package in R, the Satterthwaite approximation for degrees of freedom was applied, and F-statistics and P-values were obtained using the anova function from the lmerTest package. Multicollinearity among predictors was assessed using the variance inflation factor (VIF) function in the car package, with values exceeding 5 indicating moderate collinearity. Structural equation modeling (SEM) was constructed using the piecewiseSEM package in R to partition the direct and indirect effects of N addition on disease index through community structural variables (relative coverage, average height, and richness) and disease incidence rate. Model fit was assessed using Fisher’s C statistic and d-separation tests. Before analyzing the incidence rate and disease index, check whether the data conform to a normal distribution and satisfy the homogeneity of variance test, for data that do not meet these criteria, apply a logarithmic transformation to ensure they meet the prerequisites for analysis of variance.

## 3 Results

### 3.1 The effect of N addition on the incidence and severity of foliar fungal diseases in plant communities

With the increase in N addition rates, no significant differences in disease incidence rates were observed between the groups ranging from N0 to N16. On the contrary, the incidence rate in the N32 group was significantly higher than in the other groups (*p* < 0.01) (**Figure 1a**). Pairwise comparisons between adjacent N treatments for the disease index did not reach statistical significance (*p* > 0.05) **Figure 1b**. However, a significant positive linear trend was observed across the N gradient (R² = 0.18, *p* < 0.05; **Figures 1c, d**).

**Figure 1:**
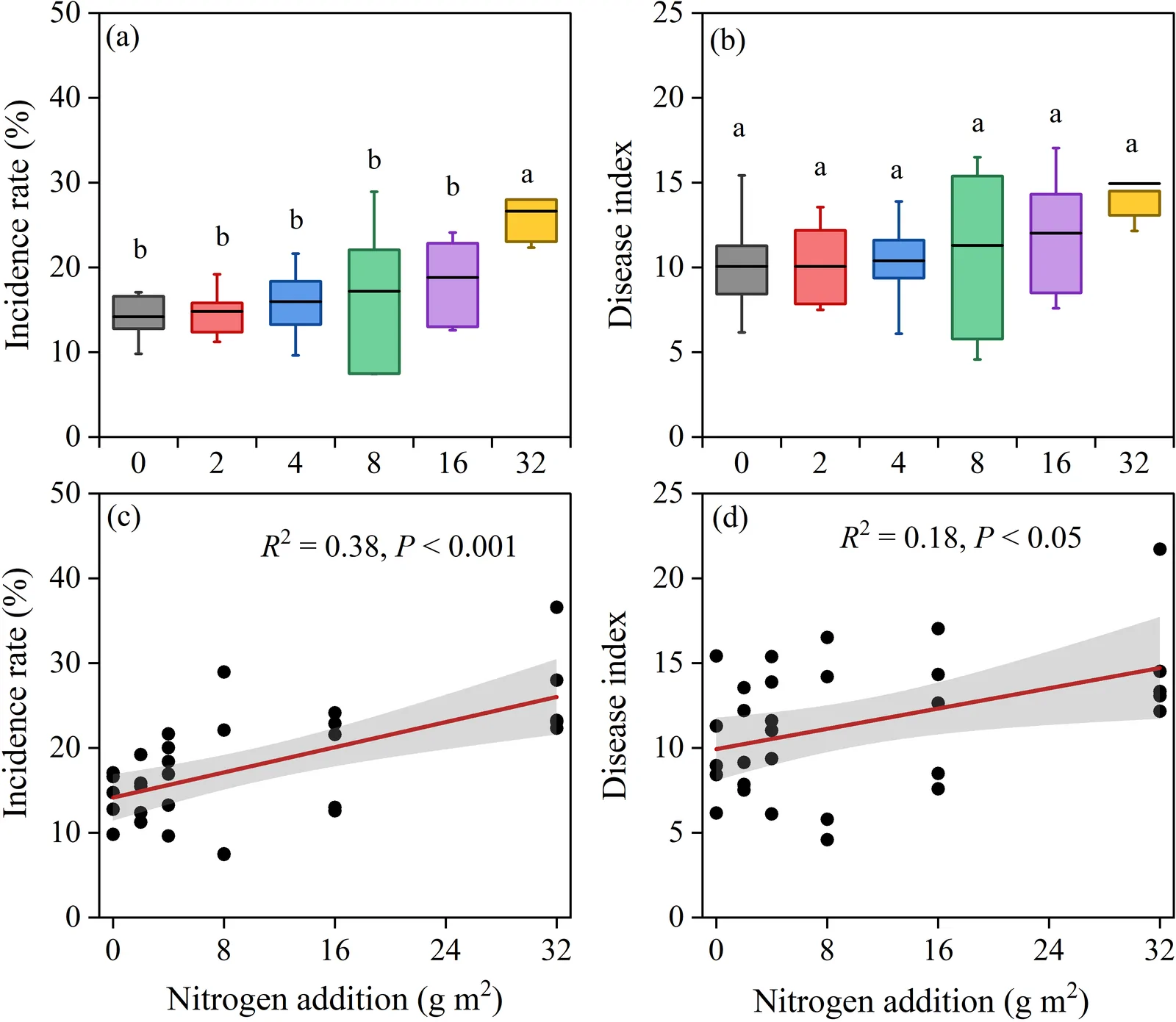
Effect of N addition on disease incidence rate **(a, c)** and disease index **(b, d)** in the plant community. Each point represents the plot-level mean of species-level values per plot (n = 5 replicate plots per treatment). Different letters indicate significant differences between different N addition treatments (*p* < 0.05). Significant relationships are indicated by solid lines. The gray shaded area represents 95% confidence intervals.

### 3.2 The effect of N addition on disease incidence and severity in different plant groups

The incidence rate and index of foliar fungal diseases increased to different degrees in all three plant groups with increasing rates of N addition, with a significant increase observed in Gramineae (*p* < 0.05), but the incidence rate and disease index of Gramineae had no significant difference among the treatments (**Figures 2a, b**). The incidence rate and the disease index in Cyperaceae were significantly higher in N8 treatment than in N0. The disease incidence of Forbs also gradually increased with the increase in the N rate, but it did not reach statistical significance (*p* > 0.05).

**Figure 2:**
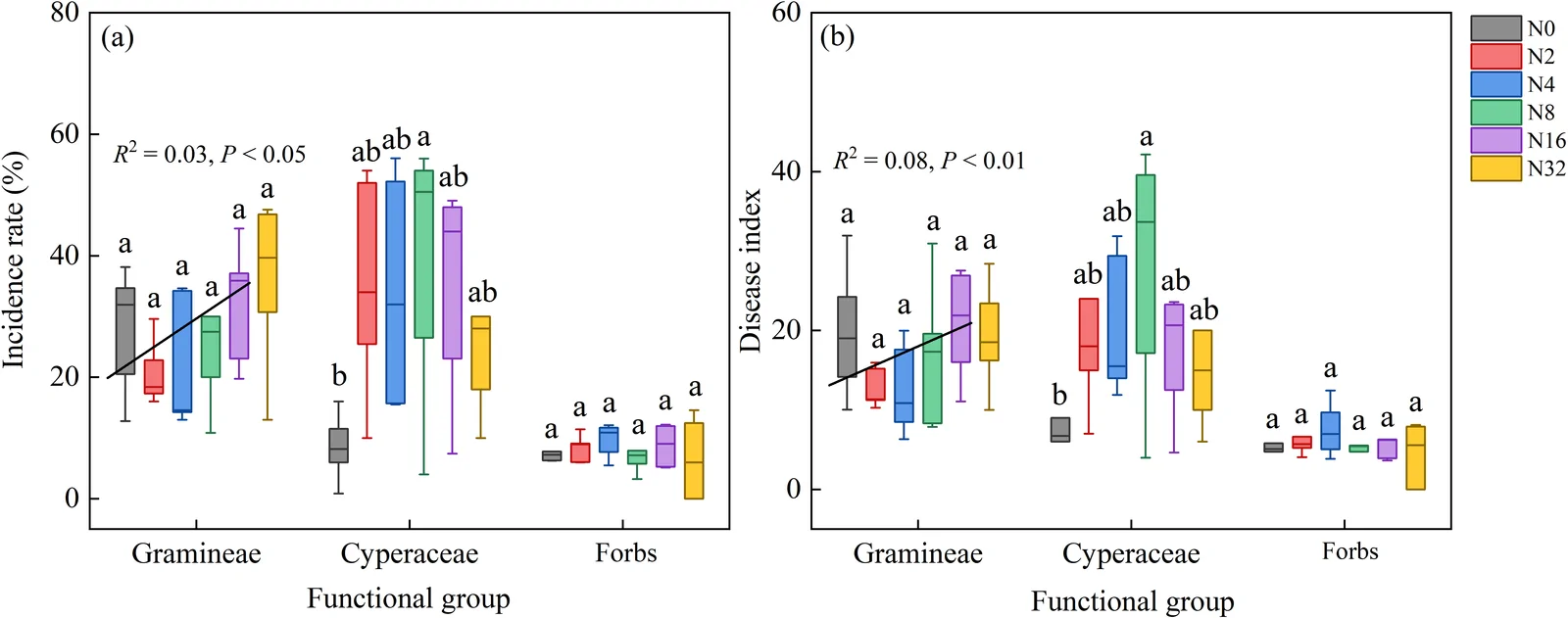
Effect of N addition on disease incidence rate **(a)** and disease index **(b)** in the different plant groups. Different letters indicate significant differences between different N addition treatments (*p* < 0.05). Significant relationships are indicated by solid lines, and those without significant linear relationships do not have regression lines. Lines within boxes represent the median.

### 3.3 The effect of N addition on the characteristics of the plant community

There were no significant differences in the average height of the plant community among N treatments (**Figures 3a, c**). However, a significant decreasing trend was observed in species richness, with N32 richness significantly lower than other treatments (**Figures 3b, d**).

**Figure 3:**
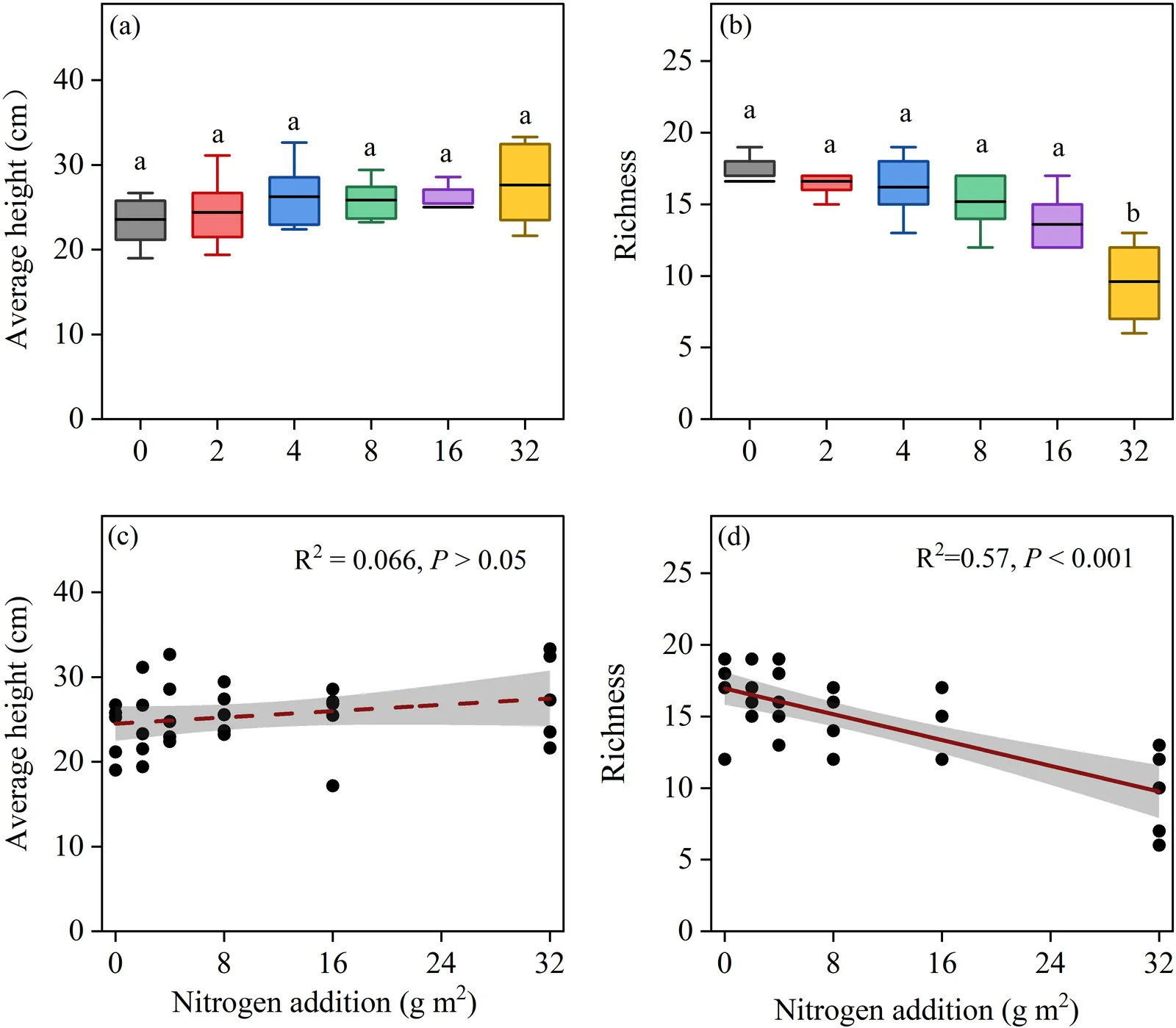
Effect of N addition on the average height **(a, c)** and richness **(b, d)** of the community. Different letters indicate significant differences between different N addition treatments (*p* < 0.05). Significant relationships are indicated by solid lines and non-significant relationships are indicated by dashed lines. The gray shaded area represents 95% confidence intervals. Each point represents the average value of the sample plot.

With increased N addition, the relative coverage of Gramineae and Cyperaceae increased significantly, while the coverage of Forbs decreased significantly (*p* < 0.001) (**Figure 4a**). Furthermore, the addition of N also significantly affected the average height (*p* < 0.01) as well as the community richness (*p* < 0.001) of Forbs (**Figures 4b, c**).

**Figure 4:**
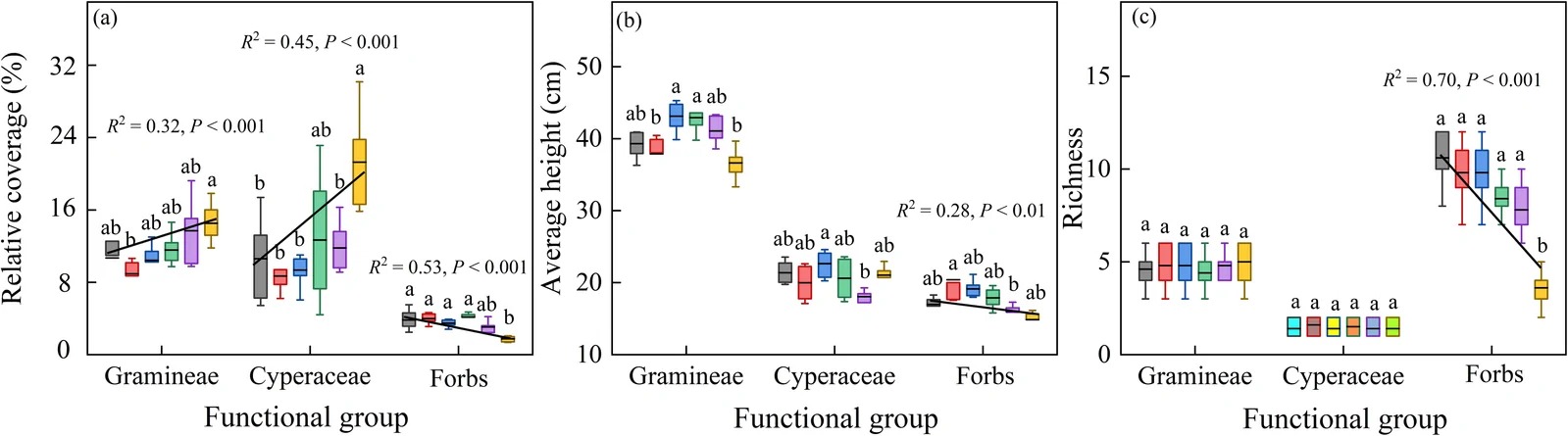
Effect of N addition on relative coverage **(a)**, average height **(b)**, and richness **(c)** of each functional group. Different letters indicate significant differences between different N addition treatments (*p* < 0.05). Significant linear relations are drawn with regression lines, and those without significant linear relationships do not have regression lines. Lines within boxes represent the median.

### 3.4 Relationships between the characteristics of the plant community and foliar fungal diseases

The incidence rate of the disease in the entire community increased with increasing relative coverage of the community (*p* < 0.001) (**Figure 5a**). The same trend was observed in each of the different plant groups, except for Cyperaceae, with the incidence rate of the disease in Gramineae and Forbs increasing significantly with increasing relative coverage (*p* < 0.01) (**Figures 5b–d**). Disease incidence rates in the entire plant community and Cyperaceae increased significantly as the average height of the community increased. On the contrary, the incidence rates in Gramineae and Forbs exhibited a decreasing trend, with a statistical significance of *p* < 0.01 observed in Forbs (**Figure 5h**). The incidence rate of disease in the entire plant community decreased significantly with increased species richness (*p* < 0.05) (**Figure 5i**).

**Figure 5:**
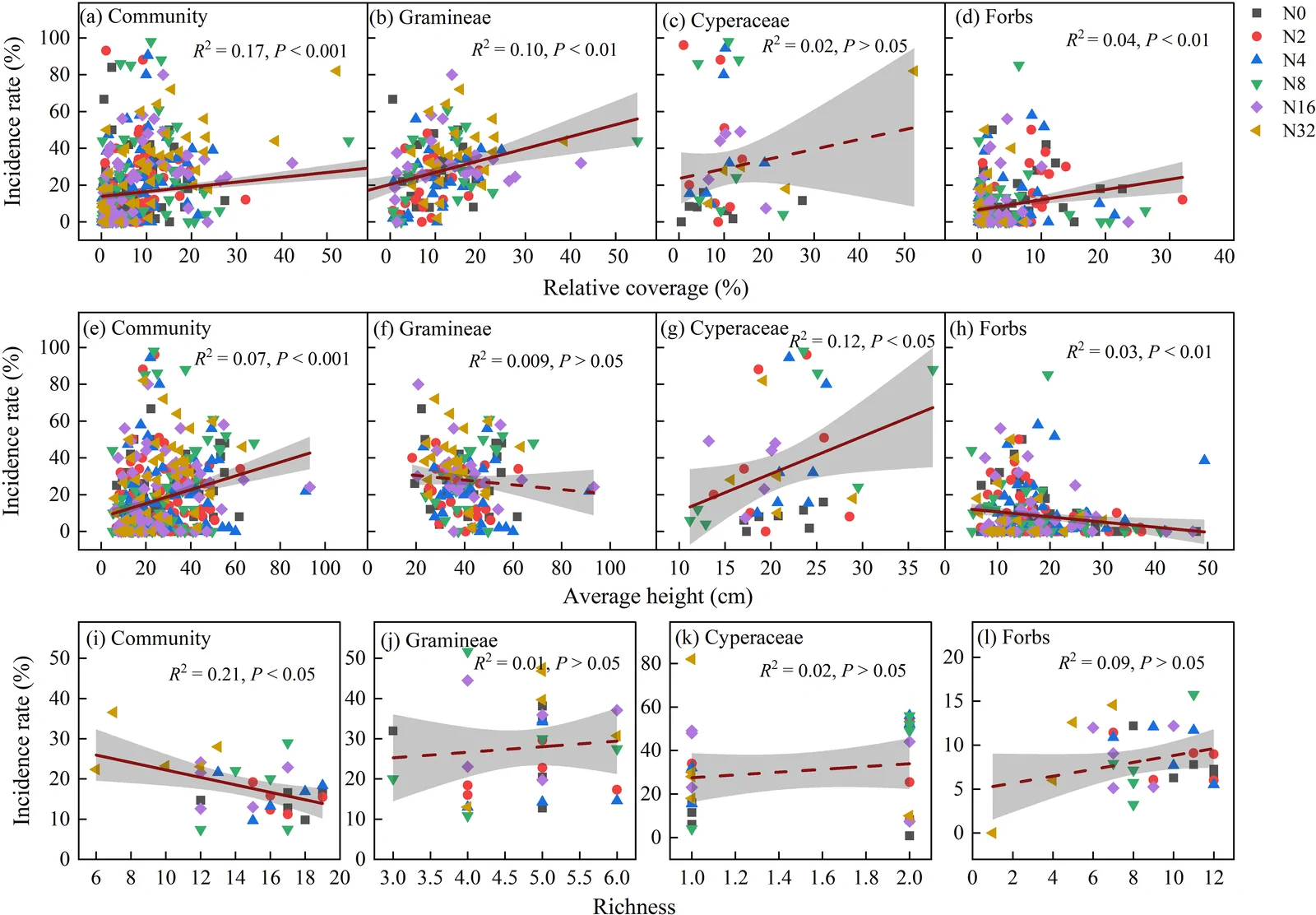
Relationships between incidence rate and relative coverage **(a–d)**, average height **(e–h)** and richness **(i–l)** of the entire plant community **(a, e, i)**, Gramineae **(b, f, j)**, Cyperaceae **(c, g, k)** and Forbs **(d, h, l)**. Significant relationships are indicated by solid lines and non-significant relationships are indicated by dashed lines. The gray shaded area represents 95% confidence intervals. The dots with different colors and shapes indicate the incidence rate of species in different N addition treatments.

Similarly to the incidence rate of the disease, the disease index of the entire plant community, as well as Gramineae and Forbs specifically, increased with relative coverage, except for Cyperaceae (**Figures 6a–d**). Furthermore, the disease severity index for the entire plant community and Forbs increased significantly with increasing community average height (**Figures 6e, h**). On the other hand, the disease index of the entire plant community declined with increasing species richness. The disease indices in three plant groups (Community, Gramineae, and Forbs) exhibited an increasing trend with richness, but did not reach a statistically significant level with respect to the differences (*p* > 0.05) (**Figures 6i–l**).

**Figure 6:**
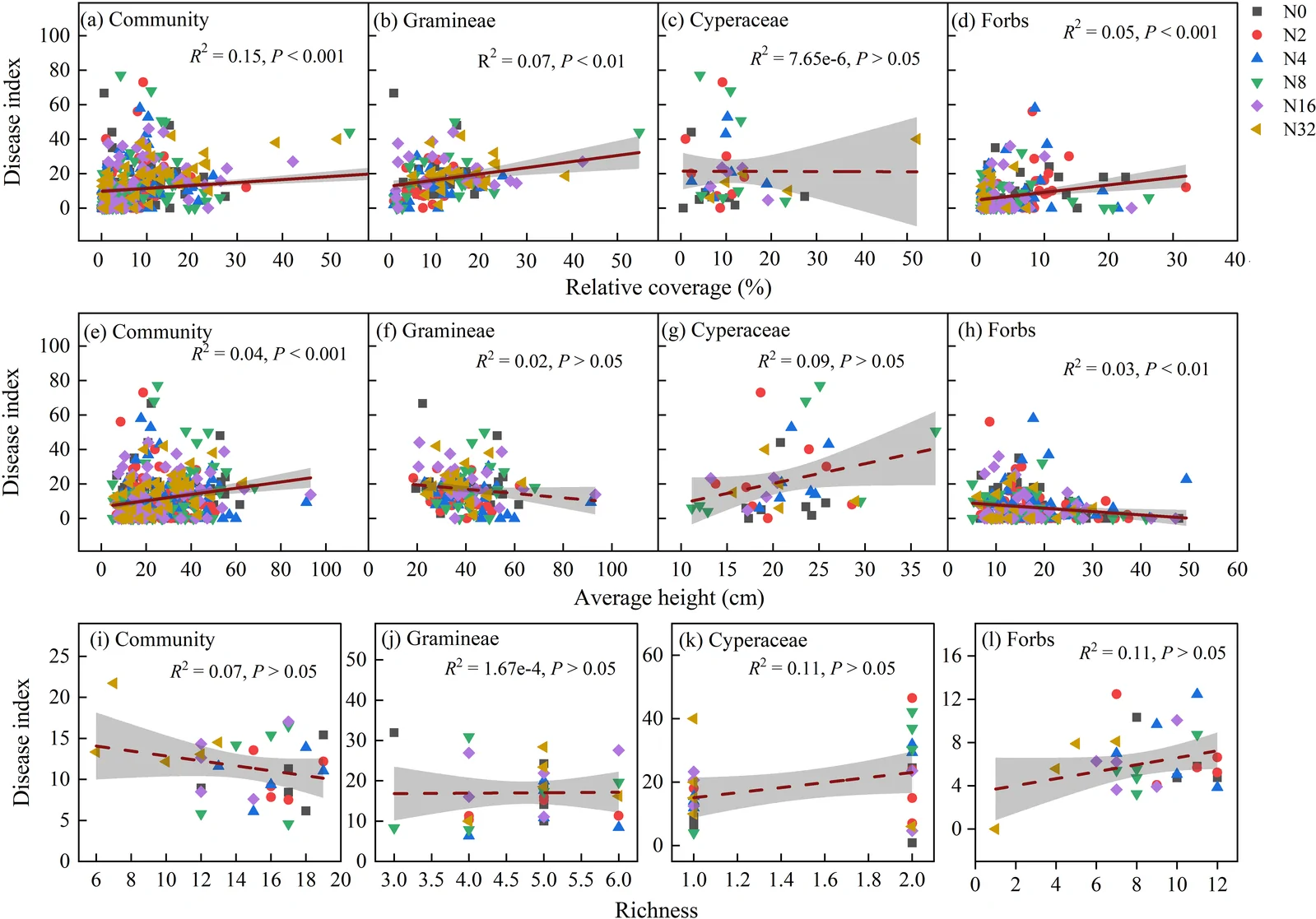
Relationships between disease index and relative coverage **(a–d)**, average height (e-h) and richness **(i–l)** of the entire plant community **(a, e, i)**, Gramineae **(b, f, j)**, Cyperaceae **(c, g, k)** and Forbs **(d, h, l)**. Significant relationships are indicated by solid lines and non-significant relationships are indicated by dashed lines. The gray shaded area represents 95% confidence intervals. The dots with different colors and shapes indicates the incidence rate of species in different N addition treatments.

The general linear mixed effect model indicated that the addition of N significantly affects the richness, relative height, and relative coverage of the plant community (*p* < 0.001) (**Table 1**), but was not significantly correlated with fungal foliar disease in the community. Multicollinearity diagnostics for the LMM predictors revealed VIF values of 6.72 for Relative_coverage and 6.84 for Richness, indicating moderate collinearity between these community structural variables. This collinearity likely inflated standard errors and contributed to the non-significant main effect of Treatment in the LMM. plant groups significantly affected foliar fungal disease, richness, relative height, and relative coverage (*p* < 0.001). The interaction between N addition and plant groups had an extremely significant positive correlation with richness and relative coverage (*p* < 0.001). plant groups played a dominant role in the occurrence of foliar fungal diseases.

**Table 1:** The general linear mixed-effects model results for the effects of N addition, plant groups and their interactions on foliar fungal diseases and plant community factors.

	N		Plant groups		N × Plant groups	
Variable	F (df_n, df_d)	P	F (df_n, df_d)	P	F (df_n, df_d)	P
Incidence rate	2.93 (1, 79)	0.09	23.58 (2, 79)	***	1.13 (2, 79)	0.33
Disease index	0.16 (1, 79)	0.69	20.15 (2, 79)	***	0.61 (2, 79)	0.55
Richness	41.41 (1, 79)	***	292.04 (2, 79)	***	45.37 (2, 79)	***
Average height	11.29 (1, 79)	**	625.32 (2, 79)	***	1.39 (2, 79)	0.26
Relative coverage	26.56 (1, 79)	***	81.66 (2, 79)	***	17.54 (2, 79)	***

* means *p* < 0.05, * * means *p* < 0.01, and * * * means *p* < 0.001. df_n means numerator degrees of freedom; df_d means denominator degrees of freedom (Satterthwaite approximation). R²m (marginal R², variance explained by fixed effects) and R²c (conditional R², variance explained by fixed and random effects combined) were calculated using the r.squaredGLMM() function (MuMIn package) in R: Incidence rate: R²m = 0.37, R²c = 0.38; Disease index: R²m = 0.32, R²c = 0.33; Richness: R²m = 0.88, R²c = 0.89; Average height: R²m = 0.93, R²c = 0.93; Relative coverage: R²m = 0.72, R²c = 0.72.

The addition of nitrogen significantly increased the incidence rate (*p* < 0.01), disease index (*p* < 0.05), and the relative coverage (*p* < 0.001) of the community, and significantly reduced the richness of plant communities (*p* < 0.001).

The SEM results (**Figure 7**) showed good fit (χ² = 4.396, df = 2, P = 0.111; Fisher’s C = 6.723, df = 4, P = 0.151; AIC = 714.26; R² = 0.88) and revealed that N addition did not exert a significant direct effect on disease index (standardized coefficient =-0.13, *p* = 0.30); rather, N addition significantly increased incidence rate (standardized coefficient = 0.66, *p* = 0.010), which in turn strongly predicted disease index (standardized coefficient = 1.060, *p* < 0.001). The indirect effect via incidence rate was 0.701, with a total effect of 0.67. Notably, the direct effects of relative coverage, average height, and richness on foliar fungal diseases were not significant in the SEM (**Figure 7**). Multicollinearity diagnostics for the LMM predictors revealed VIF values of 6.72 for relative coverage and 6.84 for richness, indicating moderate collinearity that likely inflated standard errors and contributed to the non-significant main effect of N addition in the LMM (**Table 1**).

**Figure 7:**
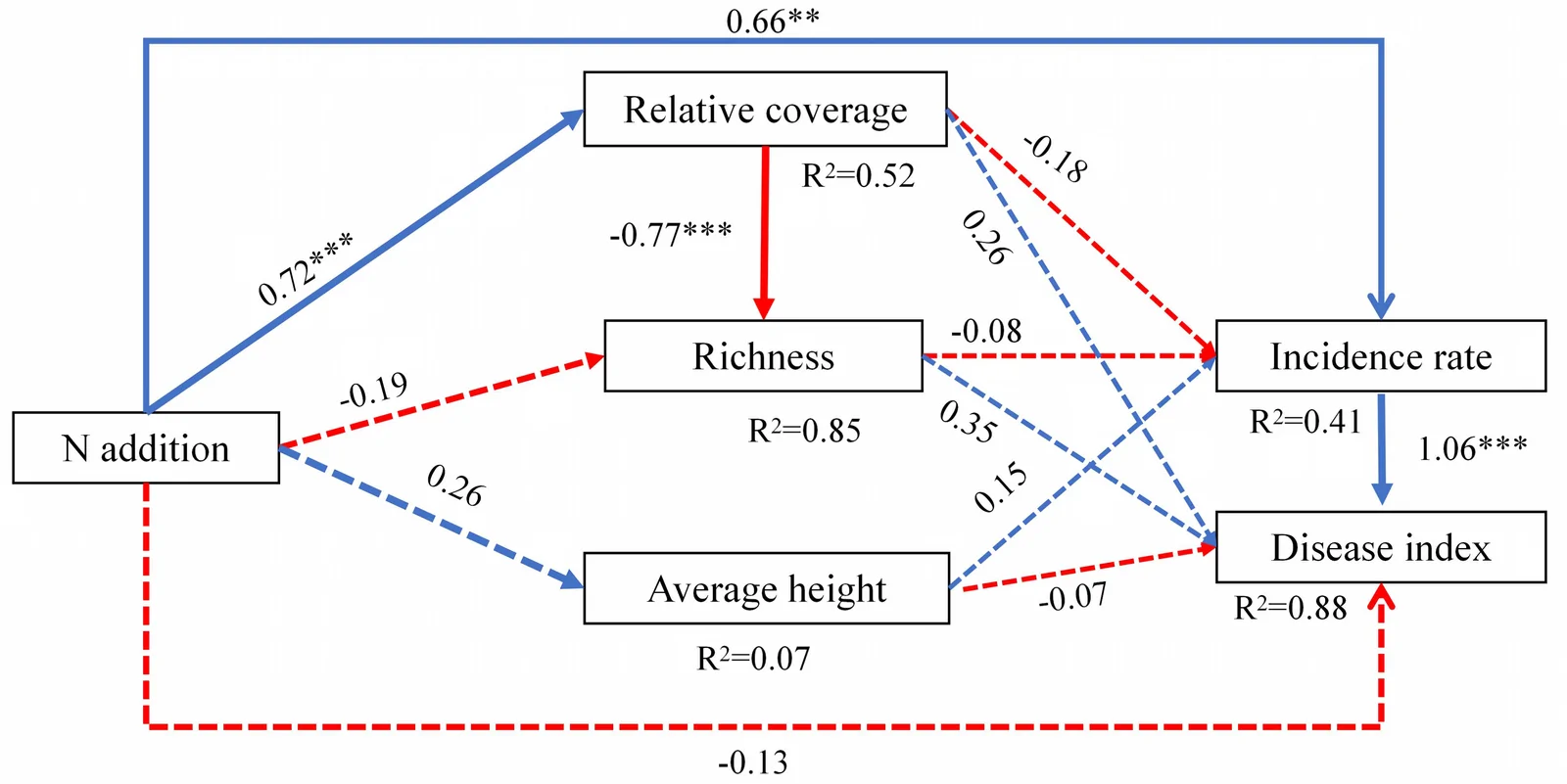
Structural equation modeling of N addition and foliar fungal diseases. Blue lines represent positive correlations, red lines represent negative correlations, solid and dashed lines indicate significant and non-significant relationships in the model, respectively, and numbers labeled by arrow lines indicate correlations (* means *p* < 0.05, ** means *p* < 0.01, *** means *p* < 0.001).

## 4 Discussion

### 4.1 Response of foliar fungal diseases to N addition

In grassland ecosystems, N significantly affects the incidence and severity of foliar fungal diseases (Cappelli et al., 2020; Godoy et al., 2018). As the rates of N addition increased, the disease incidence rate and the disease severity index showed an increasing trend in the plant community (**Figure 1**), as did the degree of plant responses to N in different plant groups. However, while there were certain distinct responses, a generally increasing trend was observed (**Figure 2**). Similarly, studies in alpine meadow ecosystems on the Tibetan Plateau have found that application of N fertilization resulted in an increase in fungal infections on plant leaves (Liu et al., 2019), which may be due to high nitrogen environments that increase leaf nitrogen content, indirectly providing a rich source of nutrients for fungal pathogens (Dordas, 2008). However, the effects of N on leaf spot disease were not static, as nitrogen enrichment was shown to increase the resilience in the function and composition of a grass community (Xu et al., 2022). Furthermore, moderate fertilization with N may reduce the severity of the disease by stimulating the compensatory growth mechanism of the plant, enhancing the tolerance of the plant to leaf spot disease, and increasing the resistance of the host plant.

To simulate the ecological effects of increasing N deposition, field experiments usually use the direct application of solid NH_4_NO_3_ to simulate N deposition (Liao et al., 2024; Tian and Niu, 2015). While this approach is standard in N addition experiments, it does not fully capture the complexity of natural atmospheric N deposition, which involves a mixture of NH_4_^+^, NO_3_^-^, and organic N compounds delivered via both dry and wet pathways at spatially and temporally variable rates. In particular, different forms of nitrogen fertilizers influence plant disease resistance differently, with NO_3_^-^ increasing disease resistance (Mur et al., 2017), while the ecological effects of NH_4_^+^ and NO_3_^-^ show significant differentiation. The two nitrogen forms differentially regulate soil pH, microbial community composition, and plant physiological responses, producing different effects (Song et al., 2007), which may affect the interaction between plants and pathogens. We acknowledge the use of solid NH_4_NO_3_ as a limitation of our experimental approach. Future research should adopt a mixed nitrogen form with proportions that match local sedimentation patterns, which would further deepen our understanding of how atmospheric nitrogen deposition affects plant diseases in alpine ecosystems.

### 4.2 Community characteristics in response to N addition

N addition can disrupt the equilibrium of competition among species, especially favoring fast-growing plants that thrive under increased nitrogen, leading to intensified competition, thus promoting species turnover and ultimately resulting in changes in the diversity and composition of plant communities (Clark and Tilman, 2008; Stevens et al., 2004). N addition altered the strength of the interactions of the species and the intrinsic growth rates, and the richness of the species decreased with increasing N addition rates (**Figure 3b**), reducing the differences in the adaptive behavior of the plant species in the community. At the same time, the formation of stable ecological niche differences was promoted (Granjelet et al., 2023). The average height of the community increased with the increase in N addition rates (**Figure 3a**) due to the fact that N-rich environments generally favor fast-growing plants (e.g., Gramineae and Cyperaceae) (Ma et al., 2020). In contrast, the average height of Forbs decreased with increasing rates of N addition (**Figure 4b**). The intensity of competition between plants is likely to increase, leading to a more pronounced effect of interspecific mechanisms (e.g., plant average height and resource use efficiency) on community structure (Ma et al., 2021; 2024). This may also result in an elevated nitrogen content in plant tissues, thus providing a richer resource for pathogens (e.g., fungi and bacteria) that rely on N as a growth substrate, which is favorable for the incidence and development of disease (Dordas, 2008). N addition can also affect community characteristics such as plant biomass, nutrient content, survival, and growth rates, and these changes are frequently associated with a decrease in conspecific host densities (Liu et al., 2022; Mitchell et al., 2003; Peters and Shaw, 1996), thus profoundly affecting grassland ecosystem diversity and stability. If N fertilization application increases disease-prone species in the community, such diversity may not be effective in reducing disease incidence (Cappelli et al., 2020).

### 4.3 Relationships between foliar fungal diseases and community characteristics under N addition

Foliar fungal pathogens indirectly shape plant community composition by impairing plant growth and health. They can promote species co-existence by suppressing dominant species through density-dependent negative effects (Bever et al., 2015; Petermann et al., 2008), thus modulating the predominant grassland species that shift grassland communities and ecosystems (Mordecai, 2011). The incidence rate of the disease and the disease index increased significantly as the relative coverage and average height of the community increased, potentially due to intensified competition for light, which may limit the access of shorter plants to resources and reduce their vigor (**Figures 4**, **5**) (Zhang et al., 2021a). Furthermore, plants with a larger canopy may provide more shade and increase humidity, which may be favorable to the survival and spread of certain pathogens, particularly those that require a moist environment for pathogen propagation (Mitchell et al., 2002). Gramineae and Forbs often have dense branches and leaves, with close contact between leaves or plants. The spores of fungal pathogens are easily physically transmitted through leaf overlap, friction, or rainwater splashing (Rottstock et al., 2014). This could also be in part attributable to taller plants, which provide physical shade to shorter plants, reducing the exposure of shorter plants to pathogen spores, and thus reducing their disease burden (**Figures 5h**, **6h**). In particular, differences in maximum plant average height are the best predictors of the prevalence and intensity of competition and facilitation (Lyu et al., 2017). In addition, differences in relative plant average height can lead to differences in the pathways and modes by which pathogens reach the plant surface, affecting the occurrence of disease (Ampt et al., 2022).

In grassland ecosystems, the effect of dilution on leaf fungal diseases due to host plant diversity is prevalent (Liu et al., 2016, 2022; Mitchell et al., 2002; Rottstock et al., 2014), resulting in a decrease in disease incidence with increasing host plant richness (Keesing et al., 2006). The same phenomenon was observed in this study (**Figure 5i**), where disease spread in a highly diverse plant community can be limited, as the prevalence of a specific pathogen can be restricted if the host plant coverage is low, reducing the likelihood that the pathogen will encounter an appropriate host and a suitable susceptible host (Liu et al., 2022; Rottstock et al., 2014; Tilman et al., 1996). On the contrary, lower plant richness and abundance in grasslands are associated with greater susceptibility and severity to disease due to reduced interspecific competition, which increases the abundance of a species in the local community and thus promotes the spread of certain species-specific diseases within the community (Ampt et al., 2018; Karunarathna et al., 2021; Mitchell et al., 2002). For single-species host plant populations, the probability that individual plants are infested with fungal pathogens increases as population density increases (Burdon and Chilvers, 1982). A reduction in the density of a specific host plant, concomitant with an increase in plant community diversity, typically leads to pathogen dilution for fungal pathogens associated with that host (Burdon and Chilvers, 1982; Collins et al., 2020). Pathogens may also exhibit amplification effects when host diversity increases, although we observed a negative correlation between plant richness and disease incidence rate (**Figure 5i**), which is consistent with the dilution model, the lack of pathogen specific data makes it impossible to attribute this model to the dilution effect itself. Many fungal pathogens can infect a wide range of different host species (Semchenko et al., 2022). In California grasslands in the United States, the severity of leaf spot disease did not show a significant positive correlation with the relative abundance of fungi due to the fact that foliar fungal pathogens are generally not host specific (Spear and Mordecai, 2018). However, it was positively correlated with the density of the perennial grass community (Kendig et al., 2021). We found an extremely high relationship between sedge richness and disease incidence (**Figure 6**). On the other hand, Liu et al. (2022) observed that suppression of leaf fungal diseases significantly altered the composition of plant community species, favoring Gramineae and Cyperaceae monocotyledonous plants. Ampt et al. (2022) found that *Paraphoma chrysanthemicola* had an increased abundance in the roots of the Forbes of the Asteraceae family, while *Slopeiomyces cylindrosporus* was abundant with grass species. Notably, the prevalence of fungal diseases is also highly dependent on interannual climate variation, particularly temperature and precipitation. Since our results are derived from only one-year observations, multi-year investigations under contrasting climatic conditions are required in future to comprehensively understand the effects of nitrogen addition on foliar fungal diseases.

The generalized linear mixed effects model showed no significant correlation between N addition and disease index in the community (**Table 1**), while the SEM detected a significant direct pathway (**Figure 7**). This difference arises because the LMM estimates a marginal net effect, whereas the SEM estimates a direct path within a causal network. Moderate collinearity between relative coverage and richness inflated standard errors in the LMM, masking the effect of N addition. Notably, the SEM revealed that N addition did not exert a significant direct effect on disease index. Instead, N addition significantly increased incidence rate, which in turn strongly predicted disease index. Effect decomposition showed that the indirect effect of N addition via incidence rate accounted for the majority of the total effect (0.67), while the direct path was negligible and non-significant. This suggests that N addition influences disease severity primarily by promoting disease incidence rather than by directly exacerbating lesion severity on already infected plants. The SEM results confirmed both hypotheses: Gramineae consistently exhibited the highest disease burden across all N levels (Hypothesis 1), and community structural traits (cover and richness) predicted disease indirectly through incidence rate (Hypothesis 2).

## 5 Conclusion

This study explored differences in the incidence and severity of foliar fungal diseases across plant groups along an N addition gradient, revealing how nitrogen enrichment affects plant diseases in natural grasslands. As supported by the study findings, excessive N addition exacerbated the risk of plant diseases in natural grasslands, with disease severity increasing at higher N addition rates. The SEM further revealed that this effect was primarily indirect: N addition promoted disease incidence, which in turn drove disease index, rather than directly exacerbating lesion severity. Plant groups differed significantly in disease incidence and disease index under varying nitrogen levels. Notably, Gramineae maintained the highest disease incidence and severity across all N addition treatments. N addition primarily influenced the occurrence of foliar fungal diseases by altering the relative coverage of plant groups and its richness. Furthermore, foliar fungal isolates exhibited a greater propensity to infest plants with greater relative coverage, especially Gramineae and Forbs. The incidence of community disease increased with increasing average height, suggesting the potential role of community structure in the spread of natural grassland diseases. This study not only improves our understanding of the relationship between N fertilization management and the incidence and severity of plant diseases in natural grasslands, but also provides important conceptual support and practical guidance for sustainable management of grasslands ecosystems, disease prevention and control, and biodiversity conservation. Future studies should explore the range of reasonable nitrogen addition to balance grassland productivity, disease prevention and control, as well as sustainable grassland use.

## Data Availability

The raw data supporting the conclusions of this article will be made available by the authors, without undue reservation.
