# Supplementary material for: The effect of nine-year nitrogen addition on foliar fungal diseases of plant groups in an alpine meadow
Source: Front Plant Sci. 2026 Sep 18;17:1899151. doi: 10.3389/fpls.2026.1899151 (PMC13632605; doi:10.3389/fpls.2026.1899151)
Supplement: Supplementary file 1 [file Table1.docx]

**Table S1.** Plant species list and information on mainly associated pathogens in the nitrogen addition experiment in this study.

| **Host** | | **Representative**  **strain** | **Number of**  **isolated strain (n)** | **GenBank accession^x^** | | | |
| --- | --- | --- | --- | --- | --- | --- | --- |
| **Family** | **Species** |  |  | **ITS** | **Most probable taxonomic matches** | **Percent identity (%)** | **Literature cited^y^** |
| *Gramineae* | *Festuca rubra* | HY2 | 1 | **PP216980** | *Cladosporium ramotenellum* (MT223790) | 100.00 | Crous et al., 2020 |
|  | *F. rubra* | HY1 | 2 | **PP216996** | *Clonostachys rosea* (MK593636) | 100.00 | Pimentel et al., 2022 |
|  | *F. rubra* | HY10 | 2 | **PP216997** | *Phaeosphaeria caricis* (MH863079) | 97.74 | Vu D et al., 2019 |
|  | *F. rubra* | HY125 | 2 | **PP217088** | *Clonostachys rosea* (MN452772) | 100.00 | Pimentel et al., 2022 |
|  | *Deschampsia caespitosa* | HY112 | 1 | **PP216982** | *Colletotrichum spaethianum* (KP127987) | 100.00 | Carvalho et al., 2016 |
|  | *D. caespitosa* | HY3 | 3 | **PP216983** | *Cladosporium herbarum* (MK919499) | 100.00 | Meyers et al ., 2019 |
|  | *D. caespitosa* | HY108 | 3 | **PP216998** | *Parastagonospora nodorum* (MH399353) | 98.57 | Spear et al., 2018 |
|  | *D. caespitosa* | HY49 | 1 | **PP216999** | *Clonostachys rosea* (MN452772) | 100.00 | Pimentel et al., 2022 |
|  | *D. caespitosa* | HY124 | 3 | **PP217092** | *Epicoccum nigrum* (MT548679) | 100.00 | Mack et al., 2021 |
|  | *Agrostis matsumurae* | HY69 | 6 | **PP216984** | *Clonostachys rosea* (MK593678) | 100.00 | Pimente et al., 2022 |
|  | *A. matsumurae* | HY127 | 2 | **PP217000** | *Parastagonospora novozelandica* (NR164453) | 98.97 | Marinfelix et al., 2017 |
|  | *A. matsumurae* | HY136 | 2 | **PP217001** | *Neoascochyta graminicola* (MH861512) | 98.28 | Vu D et al., 2019 |
|  | *A. matsumurae* | HY123 | 1 | **PP217093** | *Epicoccum nigrum* (MT548679) | 100.00 | Mackin et al., 2021 |
|  | *A. matsumurae* | HY91 | 2 | **PP217094** | *Clonostachys rosea* (MN452772) | 100.00 | Pimentel et al., 2022 |
|  | *Koeleria macrantha* | HY14 | 4 | **PP217002** | *Paraphaeosphaeria verruculosa* (JX496106) | 99.13 | Verkley et al., 2014 |
|  | *K. macrantha* | HY114 | 10 | **PP217003** | *Clonostachys rosea* (MN452772) | 100.00 | Pimentel et al., 2022 |
|  | *K. macrantha* | HY100 | 3 | **PP217095** | *Parastagonospora novozelandica* (NR164453) | 98.96 | Marinfelix et al., 2017 |
| Cyperaceae | *Carex schneideri* | HY30 | 1 | **PP216985** | *Plenodomus deqinensis* (JF740222) | 98.53 | De Gruyter et al., 2013 |
|  | *C. schneideri* | HY95 | 1 | **PP217004** | *Saccharicola bicolor* (MK102690) | 99.58 | Lu et al., 2016 |
|  | *C. schneideri* | HY90 | 3 | **PP217005** | *Stagonospora trichophoricola* (KY750315) | 99.57 | Chen et al., 2017 |

**Table S1. (Continued)**

| **Host** | | **Representative**  **strain** | **Number of**  **isolated strain (n)** | **GenBank accession^x^** | | | |
| --- | --- | --- | --- | --- | --- | --- | --- |
| **Family** | **Species** |  |  | **ITS** | **Most probable taxonomic matches** | **Percent identity (%)** | **Literature cited^y^** |
| Cyperaceae | *C. schneideri* | HY79 | 1 | **PP217006** | *Stagonospora bicolor* (MK102689) | 98.92 | Lu et al., 2016 |
|  | *C. schneideri* | HY40 | 1 | **PP217007** | *Paraphaeosphaeria verruculosa* (JX496106) | 99.12 | Verkley et al., 2014 |
|  | *C.x schneideri* | HY93 | 6 | **PP217097** | *Clonostachys rosea* (MN452772) | 100.00 | Pimentel et al., 2022 |
|  | *Kobresia myosuroides* | HY43 | 1 | **PP216987** | *Comoclathris sedi* (KP334717) | 99.35 | Wu et al., 2013 |
|  | *K. myosuroides* | HY44 | 2 | **PP216988** | *Alternaria photistica* (OL321832) | 100.00 | van der Merwe et al., 2013 |
|  | *K. myosuroides* | HY47 | 7 | **PP216989** | *Alternaria photistica* (MH861944) | 99.79 | Vohník et al., 2024 |
|  | *K. myosuroides* | HY37 | 2 | **PP217008** | *Septoriella hollandica* (MW054400) | 100.00 | Phookamsak et al., 2019 |
|  | *K. myosuroides* | HY135 | 5 | **PP217009** | *Sarocladium strictum* (KM249080) | 100.00 | Hernandez-Restrepo et al., 2017 |
|  | *K. myosuroides* | HY126 | 4 | **PP217098** | *Epicoccum nigrum* (MT548679) | 100.00 | Zhang et al., 2020 |
| *Ranunculaceae* | *Anemone rivularis* | HY78 | 3 | **PP217010** | *Truncatella angustata* (MK647988) | 100.00 | Pusz et al., 2021 |
|  | *A. rivularis* | HY118 | 3 | **PP217111** | *Didymella macrostoma* (MN187013) | 100.00 | Baturo-Ciesniewska et al., 2020 |
| *Euphorbiaceae* | *Euphorbia esula* | HY41 | 4 | **PP216994** | *Colletotrichum spaethianum* (KP127987) | 100.00 | Karimi et al., 2016 |
|  | *E. esula* | HY17 | 2 | **PP216995** | *Clonostachys rosea* (OM436895) | 100.00 | van der Merwe et al., 2021 |
|  | *E. esula* | HY27 | 3 | **PP385774** | *Didymella macrostoma* (MN187013) | 100.00 | Shafique et al., 2021 |
| Rosaceae | *Potentilla anserina* | HY9 | 2 | **PP216993** | *Boeremia linicola* (MH854943) | 100.00 | Crous et al., 2014 |
|  | *P. anserina* | HY121 | 3 | **PP217017** | *Fusarium acuminatum* (MT635295) | 100.00 | Zambounis et al., 2019 |
|  | P. *anserina* | HY38 | 2 | **PP217018** | *Truncatella angustata* (MT514378) | 100.00 | Hou et al., 2020 |
|  | P. *freyniana* | HY83 | 2 | **PP216992** | *Clonostachys rosea* (MN452772) | 100.00 | Lu et al., 2016 |
|  | P. *freyniana* | HY134 | 2 | **PP217011** | *Alternaria panax* (MH861944) | 99.78 | Vu et al., 2019 |
|  | *P. freyniana* | HY65 | 3 | **PP217012** | *Clonostachys rosea* (MN452772) | 100.00 | Adam et al., 2022 |

**Table S1. (Continued)**

| **Host** | | **Representative**  **strain** | **Number of**  **isolated strain (n)** | **GenBank accession^x^** | | | |
| --- | --- | --- | --- | --- | --- | --- | --- |
| **Family** | **Species** |  |  | **ITS** | **Most probable taxonomic matches** | **Percent identity (%)** | **Literature cited^y^** |
| Rosaceae | P. *freyniana* | HY59 | 4 | **PP217013** | *Clonostachys rosea* (MK593653) | 100.00 | Li et al., 2016 |
|  | *P. freyniana* | HY116 | 6 | **PP217014** | *Colletotrichum spaethianum* (MG888355) | 93.95 | Katoch et al., 2023 |
|  | *Potentilla freyniana* | HY8 | 3 | **PP217015** | *Subplenodomus galicola* (KY554204) | 98.12 | Crnogorac et al., 2023 |
|  | *P. freyniana* | HY122 | 4 | **PP217122** | *Epicoccum nigrum* (MT548679) | 100.00 | Pimentel et al., 2022 |
|  | *Geum aleppicum* | HY166 | 6 | **PP217220** | *Saccharicola bicolor* (MK102690) | 99.58 | Pimentel et al., 2022 |
|  | *G. aleppicum* | HY192 | 6 | **PP217329** | *Leptosphaerulina australis* (MH857343) | 100.00 | Guan et al., 2018 |
|  | *G. aleppicum* | HY196 | 9 | **PP217335** | *Fusarium acuminatum* (MT635295) | 100.00 | Wang et al., 2022 |
| Polygonacee | *Polygonum viviparum* | HY32 | 3 | **PP217020** | *Phoma haematocycla* (MH245096) | 100.00 | Valenzuela-Lopez et al., 2018 |
|  | *P. viviparum* | HY120 | 1 | **PP217123** | *Colletotrichum dematium* (OR400657) | 99.14 | Meyer et al., 2015 |
| Geraniaceae | *Geranium wilfordii* | JD168 | 7 | **PP217222** | *Nigrospora oryzae* (KT823786) | 99.79 | Oh et al., 2020 |
|  | *G. macrophylla* | HY53 | 2 | **PP217021** | *Pestalotiopsis kenyana* (MN341553) | 100.00 | Carvalho et al., 2016 |
|  | *G. macrophylla* | HY89 | 1 | **PP217126** | *Clonostachys rosea* (MN452772) | 100.00 | Crous et al., 2014 |

^x^ ITS, internal transcribed spacers 1 and 2 together with 5.8S nrDNA; sequences generated in this study are indicated in **bold**.

^y^ The cited literatures are listed at the end of the attachment..

**Literature cited**

Adam, C., & Magdalena, Ś. (2022). Species diversity of mycoplankton on the background of selected indicators of water quality in stratified mesotrophic lakes. *International Journal of Environmental Research and Public Health*, 19(20), 13298. https://doi.org/10.3390/ijerph192013298

Baturo-Ciesniewska, A., Pusz, W., & Patejuk, K. (2020). Problems, limitations, and challenges in species identifcation of Ascomycota members on the basis of ITS regions. *Acta Mycologica*, 55(1). https://doi.org/: 10.5586/am.5512

Carvalho, C. R., Wedge, D. E., Cantrell, C. L., Silva‐Hughes, A. F., Pan, Z., Moraes, R. M., Madoxx, V. L., & Rosa, L. H. (2016). Molecular phylogeny, diversity, and bioprospecting of endophytic fungi associated with wild ethnomedicinal North American plant *Echinacea purpurea* (Asteraceae). *Chemistry & Biodiversity*, 13(7), 918-930. https://doi.org/10.1002/cbdv.201500299

Chen, Z. K., C, Z., Xu, X. L., X, X., Ren JinWei, R. J., Wang WenZhao, W. W., Liu XingZhong, L. X., & Li ErWei, L. E. (2017). Trichopeptides A and B, trichocyclodipeptides A-C, new peptides from the ascomycete fungus *Stagonospora trichophoricola*. *The Journal of Antibiotics*, 70: 923-928. https://doi.org/10.1038/ja.2017.76

Crnogorac, A., Mandić, A., Godena, S., Petrović, E., & Matić, S. (2023). First report of *Alternaria alternata* causing bud and blossom blight on olive in Bosnia and Herzegovina. *New disease reports*, 48(1), e12214. https://doi.org/10.1002/ndr2.12214

Crous, P. W., Giraldo, A., Hawksworth, D. L., Robert, V., Kirk, P. M., Guarro, J., Robbertse, B., Schoch, C. L., Damm, U., Trakunyingcharoen, T. & Groenewald, J. Z. (2014). The Genera of Fungi: fixing the application of type species of generic names. *IMA fungus*, 5, 141-160. https://doi.org/10.5598/imafungus.2014.05.01.14

Crous, P. W., Wingfield, M. J., Schumacher, R. K., Akulov, A., Bulgakov, T. S., Carnegie, A. J., Jurjević, Ž., Decock, Z., Denman, S., Lombard, L., Lawrence, D. P., Stack, A. J., Gordon, T. R., Bostock, R. M., Burgess, T., Summerell, B. A., Taylor, P. W. J., Edwards, J., Hou, L. W., Cai, L., Rossman, A. Y., Wöhner, T., Allen, W. C., Castlebury, L. A., Visagie, C. M., & Groenewald, J. Z. (2020). New and interesting fungi. 3. *Fungal Systematics and Evolution*, 6, 157. https://doi.org/10.3114/fuse.2020.06.09

De Gruyter, J., Woudenberg, J. H. C., Aveskamp, M. M., Verkley, G. J. M., Groenewald, J. Z., & Crous, P. W. (2013). Redisposition of Phoma-like anamorphs in *Pleosporales*. *Studies in Mycology*, 75(1), 1-36. https://doi.org/10.3114/sim0004

Guan, Y. M., Liu, Z. B., Li, M. J., Wang, Q. X., & Zhang, Y. Y. (2018). First report of *Colletotrichum spaethianum* causing anthracnose in *Atractylodes japonica* in China. *Plant Disease*, 102(1), 239. https://doi.org/10.1094/PDIS-03-17-0443-PDN

Hernandez-Restrepo, M., Gené, J., Castañeda-Ruiz, R. F., Mena-Portales, J., Crous, P. W., & Guarro, J. (2017). Phylogeny of saprobic microfungi from southern Europe. *Studies in Mycology*, 86(1), 53-97. https://doi.org/10.1016/j.simyco.2017.05.002

Hou, L., Hernández-Restrepo, M., Groenewald, J. Z., Cai, L., & Crous, P. W. (2020). Citizen science project reveals high diversity in *Didymellaceae* (*Pleosporales*, *Dothideomycetes*). *MycoKeys*, 65, 49-99. https://doi.org/10.3897/mycokeys.65.47704

Karimi, K., Arzanlou, M., Babai-Ahari, A., & Pertot, I. (2016). Biological and molecular characterisation of *Pilidium lythri*, an emerging strawberry pathogen in Iran. *Phytopathologia Mediterranea*, 366-379. https://doi.org/10.14601/Phytopathol_Mediterr-18391

Katoch, M., Qayum, A., Kumar, A., Rani, P., & Gupta, P. (2023). *Alternaria alternata* (SDHY01/02), a fungus associated with *Lamellodysidea herbacea*: its anticancer potential and responsible constituent (s). *International Microbiology*, 26(4), 1143-1155. https://doi.org/10.1007/s10123-023-00368-8

Li, P., Wu, Z., Liu, T., & Wang, Y. (2016). Biodiversity, phylogeny, and antifungal functions of endophytic fungi associated with *Zanthoxylum bungeanum*. *International Journal of Molecular Sciences*, 17(9), 1541. https://doi.org/10.3390/ijms17091541

Lu, D., Jin, H., Yang, X., Zhang, D., Yan, Z., Li, X., Zhao, Y., Han, R., & Qin, B. (2016). Characterization of rhizosphere and endophytic fungal communities from roots of *Stipa purpurea* in alpine steppe around Qinghai Lake. *Canadian Journal of Microbiology*, 62(8), 643-656. https://doi.org/10.1139/cjm-2015-0857

Mackin, H. C., Shek, K. L., Thornton, T. E., Evens, K. C., Hallett, L. M., McGuire, K. L., DeMarche, M. L., & Roy, B. A. (2021). The ‘black box’of plant demography: how do seed type, climate and seed fungal communities affect grass seed germination?. *New Phytologist*, 231(6), 2319-2332. https://doi.org/10.1111/nph.17532

Marin-Felix, Y., Groenewald, J. Z., Cai, L., Chen, Q., Marincowitz, S., Barnes, I., Bensch, K., Braun, U., Camporesi, E., Damm, U., de Beer, Z. W., Dissanayake, A., Edwards, J., Giraldo, A., Hernández-Restrepo, M., Hyde, K. D., Jayawardena, R. S., Lombard, L., Luangsa-ard, J., McTaggart, A. R., Rossman, A. Y., Sandoval-Denis, M., Shen, M., Shivas, R. G., Tan, Y. P., van der Linde, E. J., Wingfield, M. J., Wood, A. R., Zhang, J. Q., Zhang, Y., & Crous, P. W. (2017). Genera of phytopathogenic fungi: GOPHY 1. *Studies in mycology*, 86, 99-216. https://doi.org/10.1016/j.simyco.2017.04.002

Meyer, J. B., Gallien, L., & Prospero, S. (2015). Interaction between two invasive organisms on the European chestnut: does the chestnut blight fungus benefit from the presence of the gall wasp?. *FEMS Microbiology Ecology*, 91(11), 122. https://doi.org/10.1093/femsec/fiv122

Meyers, T. R., Ferguson, J., Bentz, C., & Burton, T. (2019). Opportunistic phaeohyphomycoses in wild saffron cod *Eleginus gracilis* from waterways of Norton Sound and Toksook Bay, Alaska, USA. *Diseases of Aquatic Organisms*, 135(3), 211-226. https://doi.org/10.3354/dao03393

Oh, S. Y., Yang, J. H., Woo, J. J., Oh, S. O., & Hur, J. S. (2020). Diversity and distribution patterns of endolichenic fungi in Jeju Island, South Korea. *Sustainability*, 12(9), 3769. https://doi.org/10.3390/su12093769

Phookamsak, R., Hyde, K. D., Jeewon, R., Bhat, D. J., Jones, E. G., Maharachchikumbura, S. S., Raspé, O., Karunarathna, S. C., Wanasinghe, D. N., Hongsanan, S., & Doilom, M.(2019). Fungal diversity notes 929-1035: taxonomic and phylogenetic contributions on genera and species of fungi. *Fungal diversity*, 95, 1-273. https://doi.org/10.1007/s13225-019-00421-w

Pimentel, M. F., Srour, A. Y., Warner, A. J., Bond, J. P., Bradley, C. A., Rupe, J., Chilvers, M. I., Rojas, J. A., Jacobs, J. L., Little, C. R., Robertson, A. E., Giesler, L. J., Malvick, D., Wise, K., Tenuta, A., & Fakhoury, A. M. (2022). Ecology and diversity of culturable fungal species associated with soybean seedling diseases in the Midwestern United States. *Journal of Applied Microbiology*, 132(5), 3797-3811. <https://doi.org/10.1111/jam.15507>

Pusz, W., Baturo-Cieśniewska, A., Kaczmarek-Pieńczewska, A., Patejuk, K., & Czarnota, P. (2021). Is the survivability of silver fir under condition of strong ungulate pressure related to mycobiota of bark-stripping wounds?. *Forests*, 12(8), 976. [https://doi.org/10.3390/f12080976](https://doi.org/10.1111/jam.15507)

Shafique, S., Rafiq, R., Shafique, S., Hussain, A., Javed, A., & Mubarak, A. (2021). Novel report of Rosa indica leaf spot by *Alternaria tenussima* from Pakistan. *Pakistan Journal of Phytopathology*, 33(1), 177-182. [https://doi.org/10.33866/phytopathol.033.01.0620](https://doi.org/10.1111/jam.15507)

Spear, E. R., & Mordecai, E. A. (2018). Foliar pathogens are unlikely to stabilize coexistence of competing species in a California grassland. *Ecology*, 99(10), 2250-2259. [https://doi.org/10.1002/ecy.2427](https://doi.org/10.1111/jam.15507)

Valenzuela-Lopez, N., Cano-Lira, J. F., Guarro, J., Sutton, D. A., Wiederhold, N., Crous, P. W., & Stchigel, A. M. (2018). Coelomycetous *Dothideomycetes* with emphasis on the families *Cucurbitariaceae* and *Didymellaceae*. *Studies in mycology*, 90(1), 1-69. [https://doi.org/10.1016/j.simyco.2017.11.003](https://doi.org/10.1111/jam.15507)

van der Merwe, R., Halleen, F., van Dyk, M., Jacobs, V. G., & Mostert, L. (2021). Occurrence of canker and wood rot pathogens on stone fruit propagation material and nursery trees in the western Cape of South Africa. *Plant Disease*, 105(11), 3586-3599. https://doi.org/10.1094/PDIS-10-20-2124-RE

Verkley, G. J. M., Dukik, K., Renfurm, R., Göker, M., & Stielow, J. B. (2014). Novel genera and species of coniothyrium-like fungi in *Montagnulaceae* (*Ascomycota*). Persoonia-Molecular Phylogeny and Evolution of Fungi, 32(1), 25-51. https://doi.org/10.3767/003158514X679191

Vohník, M., & Josefiová, J. (2024). Novel epiphytic root-fungus symbiosis in the Indo-Pacific seagrass Thalassodendron ciliatum. *Mycorrhiza*, 34(5-6), 447-461. https://doi.org/10.1007/s00572-024-01161-9

Vu, D., Groenewald, M., De Vries, M., Gehrmann, T., Stielow, B., Eberhardt, U., Al-Hatmi, A., Groenewald, J. Z., Cardinali, G., Houbraken, J., Boekhout, T., Crous, P. W., Robert, V., & Verkley, G. J. M. (2019). Large-scale generation and analysis of filamentous fungal DNA barcodes boosts coverage for kingdom fungi and reveals thresholds for fungal species and higher taxon delimitation. *Studies in mycology*, 92(1), 135-154. https://doi.org/10.1016/j.simyco.2018.05.001

Wang, M. M., Crous, P. W., Sandoval-Denis, M., Han, S. L., Liu, F., Liang, J. M., Duan, W. J., & Cai, L. (2022). *Fusarium* and allied genera from China: species diversity and distribution. *Persoonia-Molecular Phylogeny and Evolution of Fungi*, 48(1), 1-53. https://doi.org/10.3767/persoonia.2022.48.01

Wu, B., Tian, J., Bai, C., Xiang, M., Sun, J., & Liu, X. (2013). The biogeography of fungal communities in wetland sediments along the Changjiang River and other sites in China. *The ISME journal*, 7(7), 1299-1309. https://doi.org/10.1038/ismej.2013.29

Zambounis, A., Thomidis, T., & Prodromou, I. (2019). First Report of *Truncatella angustata* Causing Postharvest Fruit Rots on ‘Super Chief’Apples in Greece. *Plant Disease*, 103(6), 1425. https://doi.org/10.1094/PDIS-11-18-2055-PDN

Zhang, C. B., Ren, C. H., Wang, Y. L., Wang, Q. Q., Wang, Y. S., & Weng, Q. B. (2020). Uncovering fungal community composition in natural habitat of *Ophiocordyceps sinensis* using high-throughput sequencing and culture-dependent approaches. *BMC microbiology*, 20, 1-16. https://doi.org/10.1186/s12866-020-01994-2
