# Supplementary material for: The effect of nine-year nitrogen addition on foliar fungal diseases of plant groups in an alpine meadow
Source: Front Plant Sci. 2026 Sep 18;17:1899151. doi: 10.3389/fpls.2026.1899151 (PMC13632605; doi:10.3389/fpls.2026.1899151)
Supplement: Supplementary file 2 [file Table2.docx]

**Table S2.** Distribution of pathogen genera across plant functional groups based on isolation frequency in this study.

| **Pathogen genus** | **Number of isolated strain (n)** | | | |
| --- | --- | --- | --- | --- |
|  | **Gramineae** | **Cyperaceae** | **Forbs** | **Total** |
| *Alternaria* | 0 | 9 | 2 | 11 |
| *Boeremia* | 0 | 0 | 2 | 2 |
| *Cladosporium* | 4 | 0 | 0 | 4 |
| *Clonostachys* | 23 | 6 | 12 | 41 |
| *Colletotrichum* | 1 | 0 | 11 | 12 |
| *Comoclathris* | 0 | 1 | 0 | 1 |
| *Didymella* | 0 | 0 | 6 | 6 |
| *Epicoccum* | 4 | 4 | 4 | 12 |
| *Fusarium* | 0 | 0 | 12 | 12 |
| *Leptosphaerulina* | 0 | 0 | 6 | 6 |
| *Neoascochyta* | 2 | 0 | 0 | 2 |
| *Nigrospora* | 7 | 0 | 0 | 7 |
| *Paraphaeosphaeria* | 4 | 1 | 0 | 5 |
| *Parastagonospora* | 8 | 0 | 0 | 8 |
| *Pestalotiopsis* | 2 | 0 | 0 | 2 |
| *Phaeosphaeria* | 2 | 0 | 0 | 2 |
| *Phoma* | 0 | 0 | 3 | 3 |
| *Plenodomus* | 0 | 1 | 0 | 1 |
| *Saccharicola* | 0 | 1 | 6 | 7 |
| *Sarocladium* | 0 | 5 | 0 | 5 |
| *Septoriella* | 0 | 2 | 0 | 2 |
| *Stagonospora* | 0 | 4 | 0 | 4 |
| **Total** | **57** | **34** | **72** | **163** |
